# Cognitive Determinants of Tuberculosis Preventive Behaviors in High-Risk Individuals: A Cross- Sectional Study in Iran

**DOI:** 10.34172/hpp.45929

**Published:** 2026-08-21

**Authors:** Towhid Babazadeh, Erfan Saeedi Tazekand, Sara Pourrazavi, MohammadAli Dini-Ghareoghlan, Masoomeh Gholizadeh, Sana Saedi, Khalil Maleki Chollou, Elham Davtalab Esmaeili

**Affiliations:** ^1^Department of Public Health, Sarab Faculty of Medical Sciences, Sarab, Iran; ^2^Student Research Committee, Sarab Faculty of Medical Sciences, Sarab, Iran; ^3^Research Center of Psychiatry and Behavioral Sciences, Tabriz University of Medical Sciences, Tabriz, Iran; ^4^Marand Higher Education Complex of Health, Tabriz University of Medical Sciences, Tabriz, Iran; ^5^Department of Nursing, Sarab Faculty of Medical Sciences, Sarab, Iran

**Keywords:** Tuberculosis, Health literacy, Preventive behavior, Self-efficacy, Attitude, High-risk individuals

## Abstract

**Introduction:**

Tuberculosis (TB) remains a major global public-health challenge, particularly among individuals at increased risk. This study aimed to identify the cognitive factors associated with TB preventive behaviors and assess their predictive power while controlling for sociodemographic characteristics.

**Methods:**

This cross-sectional study was conducted from April and August 2024 among 300 high-risk individuals recruited through multistage cluster sampling from four health centers in Sarab, Iran. Data were collected through researcher-developed questionnaires. Hierarchical linear regression and structural equation modeling were used to examine associations between cognitive factors and TB-preventive behaviors.

**Results:**

Cognitive factors were significantly associated with TB-preventive behaviors. Demographic characteristics explained 9.7% of the variance in preventive behaviors, and adding cognitive factors increased the explained variance to 32.4%. In the final regression model, health literacy (β=0.505, *P*=0.001) and attitude (β=0.103, *P*=0.046) emerged as the only significant cognitive predictors. SEM also showed a direct association between health literacy and preventive behaviors.

**Conclusion:**

Health literacy was the strongest determinant of TB-preventive behaviors among high-risk individuals. TB-control programs should prioritize health-literacy-based interventions and strategies that promote positive attitudes toward preventive practices in high-risk populations.

## Introduction

 Tuberculosis (TB) continues to be a significant public health issue on a global scale.^1,2^ The World Health Organization (WHO) reported that in 2022, there were 10.6 million newly diagnosed cases of TB, with approximately 1.3 million deaths attributed to the disease, including 167,000 cases of TB/HIV co-infection. Consequently, TB ranks as the second leading cause of death from infectious diseases worldwide, following COVID-19.^2,3^ Despite significant advances in diagnosis, treatment, and control, this disease appears to require a perspective beyond a purely biomedical approach.^2,4^ International reports indicate that, in addition to increasing disease burden and mortality, TB can lead to extensive social, economic, and psychological consequences for patients, families, and health systems.^5-7^

 During the late 1980s, tuberculosis incidence, which had been declining in many regions, was significantly reversed by the emergence of the HIV pandemic.^8^ HIV-induced immunosuppression increased susceptibility to active TB infection, reduced treatment effectiveness, and contributed to increased transmission within communities.^8^ In addition, the dual burden of TB and HIV facilitated treatment complications and contributed to the emergence and spread of drug-resistant tuberculosis strains.^9,10^ More recently, the COVID-19 pandemic has further disrupted TB control programs globally.^11^ Due to clinical similarities between TB and COVID-19, particularly chronic cough, fever, and respiratory symptoms, many TB cases were misdiagnosed or delayed in diagnosis, leading to interruptions in treatment services and surveillance systems.^11^ Collectively, both HIV and COVID-19 have significantly altered TB epidemiological trends and undermined progress in TB control efforts over time.

 In many low- and middle-income countries, including Iran, social conditions such as poverty, food insecurity, poor housing, overcrowding, and undernutrition, along with behavioral factors significantly influence TB transmission.^1^ Undernutrition and food insecurity directly weaken immune system function, increasing susceptibility to TB infection and worsening disease outcomes.^12^ These poverty-related determinants often cluster together, creating environments that facilitate ongoing transmission and delayed diagnosis.^12^ Neglecting these factors can reduce the effectiveness of disease control programs.^2^

 Preventive behaviors, including timely visits to health centers, participation in screening programs, adherence to preventive principles, and compliance with health recommendations, are fundamental to controlling this disease.^4,13^ However, non-adherence to these behaviors remains a major barrier to effective disease control. For example, an untreated individual with active pulmonary tuberculosis may transmit the infection to approximately 10–15 close contacts per year.^14^ Lack of awareness among patients and their household contacts regarding symptoms and transmission routes can therefore lead to delayed diagnosis and sustained community transmission.^7,15^

 In recent decades, increasing attention has been given to cognitive and psychosocial determinants of health behaviors.^7,16^ Theoretical frameworks such as the Health Belief Model, Social Cognitive Theory, and the Theory of Planned Behavior suggest that preventive behaviors are shaped by complex interactions among knowledge, attitudes, perceived risks, and self-regulatory capacities.^16^ According to these theories and models, factors such as awareness of the disease, attitudes toward health outcomes, perceived ability to perform recommended behaviors (self-efficacy), and the ability to understand and use health information play a decisive role in the adoption and maintenance of preventive behaviors.^16^

 The literature has shown that TB-related knowledge can increase individuals’ sensitivity to the risk of the disease and the necessity of its prevention,^1,4,13^ although increased knowledge alone does not necessarily lead to behavior change.^16^ Individuals’ attitudes toward TB, including perceptions of disease severity, associated social stigma, and beliefs about the effectiveness of preventive measures, can also play an important role in decision-making.^13,17^ In addition to knowledge and attitude, self-efficacy, as a central construct in Social Cognitive Theory, plays a decisive role in translating cognitive perceptions into actual behavior.^16^ Self-efficacy pertains to a person’s belief in their capability to plan and implement the necessary actions to handle particular situations.^16^ Research indicates that those with elevated self-efficacy are more inclined to engage in and sustain preventive behaviors.^17^ In relation to tuberculosis prevention, self-efficacy signifies the degree to which a person is convinced they can perform actions like seeking timely medical advice for symptom assessment, adhering to prolonged treatment, following preventive guidelines, and adhering to health recommendations, even when faced with obstacles such as medication side effects, financial challenges, or social stigma.^7^ Evidence suggests that individuals with higher self-efficacy are not only more likely to initiate preventive behaviors but are also more persistent in maintaining them;^16^ therefore, this construct can be considered one of the most important predictors of preventive behaviors in patients with TB.^7^

 Health literacy, as another key determinant of health behavior, has a profound impact on decision-making, preventive behaviors, and individual and public health outcomes.^18^ Health literacy refers to an individual’s ability to seek, understand, evaluate, and apply health information in real-life situations.^18^ In the context of TB prevention, an individual’s health literacy indicates the extent to which they can understand health messages, distinguish accurate from inaccurate information, and adhere to preventive recommendations. However, the level of health literacy is not uniform across populations, and factors such as age, educational level, and socioeconomic status can influence it.

 Despite the importance of cognitive determinants, existing evidence on TB preventive behaviors has several limitations. Many studies have focused on isolated cognitive factors without accounting for sociodemographic influences, making it difficult to determine the independent contribution of psychological constructs. In addition, limited research has compared the relative predictive power of different cognitive variables within integrated models, particularly in middle-income countries such as Iran.

 Strengthening TB preventive behaviors requires targeted, population-specific strategies rather than general recommendations. Such interventions should primarily focus on high-risk groups, including individuals with chronic diseases (e.g., diabetes, kidney disease, and pulmonary disorders), immunocompromised patients, household contacts of TB cases, and populations living in overcrowded or low-income settings.^19^

 At the individual level, increasing awareness of key TB symptoms, particularly persistent cough lasting more than two weeks, unexplained weight loss, night sweats, and fever, can facilitate early healthcare seeking and timely diagnosis.^7,15^ Additionally, improving knowledge of TB transmission routes can enhance adherence to preventive practices and reduce community spread.^7,15^

 Accordingly, the present study was designed and conducted to determine the cognitive factors influencing TB preventive behaviors and to assess the predictive power of each of these factors while controlling for demographic variables. The findings of this study can play an important role in enhancing the effectiveness of disease control programs and achieving public health goals.

 Based on the literature and relevant health behavior theories, a conceptual model was developed to explain the relationships among educational attainment, tuberculosis-related knowledge, health literacy, self-efficacy, attitude, and TB preventive behaviors (Figure 1).

**Figure 1:**
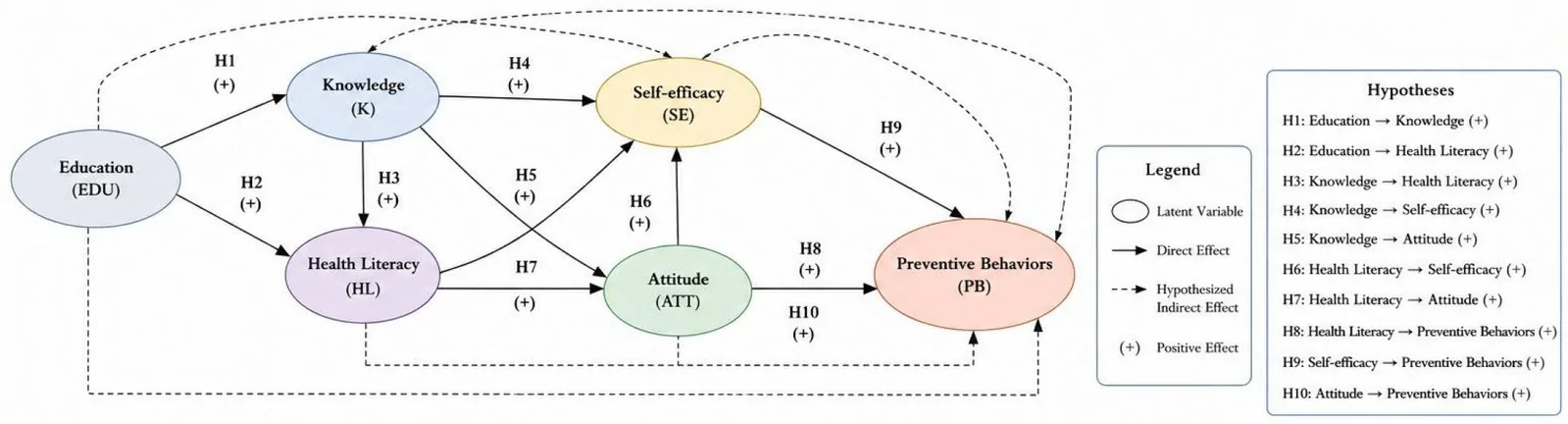
Hypothesized conceptual model of cognitive determinants of TB preventive behaviors

## Materials and methods

###  Study Design

 This was a cross-sectional study conducted from April to August 2024. The study was conducted in Sarab city, which has four health centers. The study population included patients with high-risk conditions for tuberculosis, including diabetes, lung disease, kidney disease, heart disease, smokers, and people with immunosuppressive medications (e.g., cancer patients). HIV-positive individuals were not present in the final study sample; therefore, this condition was not reported as a separate category in Table 1. Eligibility criteria included having at least one of the mentioned high-risk conditions and willingness to participate in the study. The exclusion criteria were refusal to participate and incomplete questionnaire responses. A multistage cluster sampling method was used to recruit 300 participants. The sample size was calculated based on the formula $\frac{z^{2}s^{2}}{d^{2}}$ and data from a similar study.^20^ Each of the four health centers in Sarab city was treated as a separate cluster. Eligible participants were randomly selected from these centers using health records.

**Table 1:** The Relationship Between Demographic Variables and TB Preventive Behaviors Among Participants (n = 300)

**Demographic Variables**	**Items**	**n (%)**	**Me±SD**	***P*** -value*
Gender	Male	126 (42)	21.48 ± 5.60	0.997
	Female	174 (58)	21.01 ± 5.68	
Age groups	50 ≥	149 (49.7)	21.21 ± 6.60	0.468
	50 <	151 (50.3)	21.21 ± 5.18	
Level of education	Illiterate & Elementary	94 (31.3)	19.54 ± 5.50	0.001
	Secondary school and High school	93 (31.0)	21.03 ± 4.87	
	Diploma and higher	113 (37.7)	23.34 ± 5.83	
Marriage status	Married	224 (74.7)	20.98 ± 5.62	0.001
	Single **	76 (25.3)	20.22 ± 5.57	
Income	Less than 10	93 (31)	22.83 ± 5.21	0.001
	10 to 15	109 (36.3)	21.06 ± 6.17	
	More than 15	98 (32.7)	19.83 ± 5.06	
High-risk conditions	diabetes	82 (27.3)	20.74 ± 5.43	0.063
	lung disease	53 (17.7)	21.96 ± 5.33	
	Heart disease	71 (23.7)	22.57 ± 5.46	
	kidney disease	31 (10.3)	20.48 ± 6.08	
	People with immunosuppressive drugs ٭٭٭	63 (21.0)	20.03 ± 5.86	
	Smoking			
	Yes	90 (30.0)	20.08 ± 5.37	0.351
	No	210 (70.0)	21.69 ± 5.68	

^*^ Chi-square test ^**^ single, divorced and widow

###  Data Collection Instruments

 The instruments utilized for data collection were questionnaires developed by the researchers. To ensure the content validity of these instruments, a panel of experts, comprising four scholars in health education and three physicians specializing in infectious diseases, conducted an oral review and assessment of the questions. They evaluated the appropriateness and relevance of the items for the participants, the response format, and confirmed that the items accurately represented the constructs. The feedback from the expert panel was instrumental in revising and modifying the tools, which were subsequently pilot tested with a sample of study participants to evaluate their effectiveness. The pilot study aimed to assess the utility of the instruments and to identify any issues or advantages related to their design.

 The initial draft was created after discussions with the research team. The questionnaire underwent pilot testing with 25 individuals who met the study’s inclusion criteria. The data collected were utilized to estimate the internal consistency of the scales, employing Cronbach’s coefficient alpha. Additionally, the content validity of the scales was confirmed. It is important to note that this pilot sample was not included in the final sample. For the data collection process, a questionnaire was designed to capture demographic characteristics (age, gender, education level, marital status, and monthly income), as well as knowledge, attitude, self-efficacy regarding TB health literacy, and TB preventive behaviors.

***The Tuberculosis Knowledge Questionnaire (TKQ):*** A 15-item scale developed to measure knowledge about tuberculosis. Respondents selected one of three options: “correct,” “incorrect,” or “I don’t know.” Correct answers received a score of 1, and incorrect answers received a score of 0. An example item is: “Tuberculosis is a contagious disease.” The theoretical range was 0–15, with higher scores indicating greater knowledge (Cronbach’s α = 0.86).

***The Tuberculosis Attitude Questionnaire (TAQ):*** A 13-item scale to measure attitudes toward TB preventive behaviors, using a four-point Likert scale (3 = totally agree, 2 = agree, 1 = disagree, 0 = totally disagree). Example items include: “Tuberculosis screening can be easily done at health centers” and “I think regular medical checkups can help prevent tuberculosis.” The theoretical range was 0–39, with higher scores indicating more positive attitudes (Cronbach’s α = 0.90).

***The Tuberculosis Prevention Self-Efficacy Scale (TPSS):*** An 18-item researcher-prepared scale to measure self-efficacy toward tuberculosis prevention. Respondents selected one of three options: “I can,” “I can’t,” or “I don’t know.” Example items include: “I can use a mask when I am in crowded places” and “I can avoid close contact with people who have tuberculosis.” The theoretical range was 0–18, with higher scores indicating greater self-efficacy (Cronbach’s α = 0.83).

***The Tuberculosis Prevention Health Literacy Scale (TPHLS):*** A 22-item scale to assess health literacy for tuberculosis prevention, using a five-point Likert scale (1 = strongly disagree, 2 = disagree, 3 = no idea, 4 = agree, 5 = strongly agree). Example items include: “I can find healthcare resources from hospitals, clinics, etc., if I need information about tuberculosis” and “I can understand the information provided by healthcare professionals about tuberculosis prevention.” The theoretical range was 22–110, with higher scores indicating better health literacy (Cronbach’s α = 0.92).

***The Tuberculosis Preventive Behaviors Scale (TPBS):*** An 11-item instrument with a four-point scale (0 = strongly disagree, 1 = disagree, 2 = agree, 3 = strongly agree) to measure preventive behaviors such as: “I am actively involved in education about tuberculosis” and “I have regular chest X-rays every year.” The theoretical range was 0–33, with higher scores indicating better preventive behaviors (Cronbach’s α = 0.77).

 A questionnaire for demographic characteristics (age, gender, education level, marital status, and monthly income).

 Content validity was confirmed by a panel of experts comprising four scholars: one health education specialist and three physicians specializing in infectious diseases. The experts reviewed and assessed the questions by evaluating the appropriateness and relevance of the items, response format, and representativeness, which were then pilot tested with a sample of patients. Following consultation with the experts, the instruments were pilot tested with 25 individuals meeting the inclusion criteria. The data were used to estimate the internal consistency of the instruments using Cronbach’s coefficient alpha.

###  Data Collection Procedure

 Participants were invited by telephone. Upon arrival at the health center, they were informed about the study objectives and asked to sign a written informed consent form before participation. The questionnaires were administered in a private consultation room within the health center. To ensure participant comfort, particularly given the sensitivity of the topic and cultural considerations, all interviews were conducted by three trained interviewers.

###  Statistical Analysis

 Statistical analyses were performed utilizing SPSS (SPSS Inc, Chicago, IL, USA) version 21. The data are expressed as mean ± SD for quantitative variables and as frequency (percentage) for qualitative variables. Missing data were minimal (< 5%) and were handled using listwise deletion (complete case analysis). The Kolmogorov–Smirnov test was employed to evaluate the normality of data distribution. Hierarchical linear regression analysis was executed in two phases. Initially, sociodemographic variables were incorporated into the model using the enter method. In the subsequent phase, sociodemographic variables were added alongside cognitive determinants (knowledge, attitude, self-efficacy, and health literacy) through hierarchical linear regression. The significance threshold for entry variables in the hierarchical linear regression model was established at *P* < 0.05. The coefficient of determination (R2) was utilized to evaluate the model’s goodness of fit, quantifying the proportion of variance in the dependent variable that can be predicted from the independent variables. The assumptions underlying regression were validated through tests for multicollinearity, normality, and influential data points. The significance level was set at α = 0.05.

 To further investigate the pathways connecting educational attainment, tuberculosis-related knowledge, health literacy, self-efficacy, attitude, and preventive behaviors, structural equation modeling (SEM) was carried out using Stata. The proposed model was formulated based on the study’s conceptual framework and the findings from the hierarchical regression analysis. Model fit was assessed using various indices, including χ^2^/df, Comparative Fit Index (CFI), Tucker–Lewis Index (TLI), Root Mean Square Error of Approximation (RMSEA), and Standardized Root Mean Square Residual (SRMR). CFI and TLI values exceeding 0.90, RMSEA values below 0.08, SRMR values under 0.08, and χ^2^/df values less than 3 were regarded as indicative of an acceptable model fit.

###  Ethical Approval

 Ethical approval for this study was obtained by the Ethics Committee of the Sarab Faculty of Medical Sciences (Ethics Code: IR.SARAB.REC.1402.011). Written informed consent was obtained from all participants prior to their inclusion in the study.

## Results

 A total of 300 high-risk individuals participated in the study. The majority of the study participants were female (58%) and in the age group of more than 50 years (50.3%). The majority of the people had education level of diploma and higher (37.7%). Also, the majority of participants had diabetes, and 70% reported being non-smokers. Based on results, there was statistically a significant association between TB preventive behaviors with education level, marriage status and monthly income (*P*-value < 0.05). Table 1 displays the details of demographic characteristics and their relationship with TB preventive behaviors.

Table 2 shows the dimensions of cognitive variables and TB Preventive Behaviors as determined by the Pearson correlation test. All domains had a significant positive correlation with TB preventive behaviors, according to this test. The strongest correlation between health literacy and TB preventive behaviors was discovered (r = 0.545).

**Table 2:** Bivariate Correlation Between Cognitive Variables, and TB Preventive Behaviors (n = 300)

**Variables**	**1**	**2**	**3**	**4**	**5**
1 = Knowledge	1				
2 = Attitude	0.223 ٭	1			
3 = TB Health literacy	0.262٭	0.445٭	1		
4 = Self-efficacy	0.167٭	0.459٭	0.619٭	1	
5 = TB Preventive behaviors	0.247٭	0.270٭	0.545٭	0.335٭	1

*Correlation is significant at the 0.05 level (two-tailed)

 The most important factors and changes in TB preventive behaviors were predicted using hierarchical linear regression analysis (Table 3). The variables were able to predict 9.7 percent changes in TB preventive behaviors in the first step, which included all demographic variables. Among demographic variables education level was the strongest predictor of the TB preventive behaviors. In the second step of hierarchical regression analysis, cognitive variables were included in the model to predict TB preventive behaviors in addition to demographic variables. The predictability increased to 32.4 % with the addition of variables in this step. Health literacy, amongst variables, was significant predictors of TB preventive behaviors (β = 0.505).

**Table 3:** Hierarchical Linear Regression of TB Preventive Behaviors onto Demographic Variables and Cognitive Variables (n = 300)

**Step/variables**	β^**^ (Step 1)	***P*** -value*	β^**^ (Step 2)	***P*** -value*
(1) Gender	0.066	0.282	0.071	0.186
Age groups	0.035	0.596	0.045	0.358
Education level	-0.278٭	0.001	-0.868	0.243
Marital status	0.074	0.237	0.043	0.438
Income	-0.111	0.093	0.066	0.259
(2) Knowledge			0.026	0.656
Attitude			0.103	0.046٭
Health literacy			0.505	0.001٭
Self-efficacy			0.018	0.779
R^2^	0.097	0.001	0.324	0.001

^*^ *P* < 0.05 ^**^β is the standardized coefficient

 SEM was performed to examine the hypothesized pathways among educational attainment, tuberculosis-related knowledge, health literacy, self-efficacy, attitude, and tuberculosis preventive behaviors. The final model demonstrated an excellent fit to the observed data (χ^2^ = 4.68, df = 5, *P* = 0.456; χ^2^/df = 0.94; CFI = 1.000; TLI = 1.000; RMSEA = 0.001; SRMR = 0.023).

 Educational attainment positively predicted tuberculosis-related knowledge (β = 0.261, *P* < 0.001) and health literacy (β = 0.331, *P* < 0.001). Knowledge was positively associated with health literacy (β = 0.355, *P* < 0.001), self-efficacy (β = 0.228, *P* < 0.001), and attitude toward tuberculosis preventive behaviors (β = 0.134, *P* = 0.030). Health literacy was positively associated with self-efficacy (β = 0.516, *P* < 0.001), attitude (β = 0.202, *P* = 0.001), and preventive behaviors (β = 0.517, *P* < 0.001). Attitude was also significantly associated with preventive behaviors (β = 0.114, *P* = 0.022), whereas self-efficacy did not show a significant direct effect on preventive behaviors (β = −0.003, *P* = 0.962) (Table 4). Overall, the SEM findings highlighted health literacy as the central construct within the model, showing a significant direct association with tuberculosis preventive behaviors and meaningful associations with other cognitive constructs. Educational attainment was linked to tuberculosis-related knowledge and health literacy within the hypothesized model. Furthermore, knowledge was associated with health literacy, self-efficacy, and attitude, suggesting possible explanatory pathways that should be confirmed in future studies using formal mediation analysis.

**Table 4:** Structural Path Coefficients

**Path**	**β**	***P*** **-value**
Education → Knowledge	0.261	< 0.001
Education → Health Literacy	0.331	< 0.001
Knowledge → Health Literacy	0.355	< 0.001
Knowledge → Self-efficacy	0.228	< 0.001
Knowledge → Attitude	0.134	0.030
Health Literacy → Self-efficacy	0.516	< 0.001
Health Literacy → Attitude	0.202	0.001
Health Literacy → Preventive Behaviors	0.517	< 0.001
Self-efficacy → Preventive Behaviors	-0.003	0.962
Attitude → Preventive Behaviors	0.114	0.022

**Figure 2:**
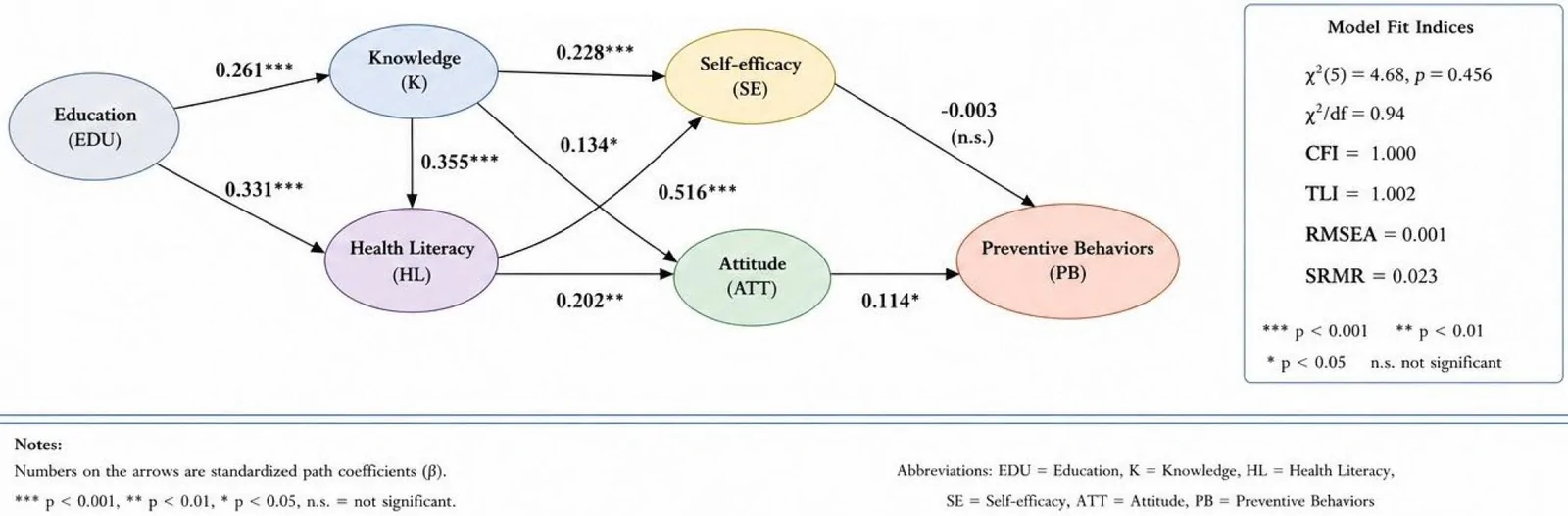
Final structural equation model of TB preventive behaviors

## Discussion

 The study’s findings supported the validity of some demographic and cognitive factors in predicting TB preventive behaviors. Specifically, health literacy, followed by attitude, was a strong predictor of preventive behaviors against TB. In addition, some sociodemographic variables showed associations with TB preventive behaviors in bivariate analyses; however, income was not statistically significant in the final multivariable regression model after adjustment for cognitive factors. Overall, the results of this study emphasize the importance of the role of cognitive factors, particularly the ability to understand and apply health information, in promoting TB-related preventive behaviors.

 The SEM analysis provided additional insight into the pattern of associations among cognitive factors and TB preventive behaviors. Beyond identifying health literacy as the strongest direct predictor of preventive behaviors, the SEM findings suggested that health literacy occupies a central position within the cognitive framework of TB prevention. Higher educational attainment was associated with greater TB-related knowledge and higher health literacy, while knowledge was positively associated with health literacy, self-efficacy, and attitude. In addition, health literacy was positively associated with attitude and preventive behaviors. These findings suggest possible pathways through which educational attainment and TB-related knowledge may contribute to preventive behaviors via health literacy and attitude. However, because formal mediation analysis was not conducted, these pathways should be interpreted as exploratory and hypothesis-generating rather than confirmatory evidence of mediation.

 Importantly, the implications of these findings should be interpreted in the context of a high-risk population. In the present study, participants included individuals with underlying conditions such as diabetes, chronic lung disease, kidney disease, cardiovascular disease, smoking, and immunosuppressive conditions. These subgroups are biologically and socially more vulnerable to tuberculosis due to compromised immunity, frequent healthcare contact, and higher exposure to healthcare environments.^19^

 In such high-risk groups, cognitive determinants of TB preventive behaviors may operate more strongly and differently compared to the general population. For example, individuals with chronic diseases often have more frequent interactions with healthcare systems, which may increase their exposure to health information and enhance the role of health literacy in shaping preventive behaviors.^21^ Conversely, disease burden, treatment complexity, and competing health priorities may weaken the direct effect of constructs such as self-efficacy,^22^ as observed in our findings where self-efficacy did not show a significant direct association with preventive behaviors.

 Moreover, in immunocompromised individuals and patients with chronic conditions, perceived susceptibility to TB is typically higher,^9,10^ which may amplify the influence of attitudes and health literacy on behavioral decisions. However, this same vulnerability may also create psychological fatigue or information overload, making simplified and actionable health communication particularly important.^18,23,24^ These factors suggest that cognitive determinants in high-risk populations are not only more influential but also more dependent on the accessibility and usability of health information.

 The findings of this study showed that the level of education has a significant relationship with TB preventive behaviors. The literature has shown that education plays important role in increasing awareness and the ability to understand health information.^18,23,25^ Individuals with higher levels of education usually have greater access to information resources, seek more health information, and have a better ability to understand health messages, recognize disease symptoms, and understand the modes of transmission and prevention of TB.^26^ In addition, higher education is associated with improved health literacy, decision-making skills, and personal health responsibility, which can contribute to better adoption and implementation of preventive behaviors.^18,26^ Previous studies in TB control have also shown that individuals with higher education have better awareness and attitudes toward this disease and are more likely to engage in preventive behaviors such as timely visits to health centers, adherence to hygiene principles, and treatment follow-up.^27,24^ Therefore, it seems that education level plays an important role in strengthening preventive behaviors related to TB by increasing health knowledge and improving the ability to use health information.

 According to the literature, married people have reported that they typically have more social and emotional support, which can play an important role in adherence to preventive behaviors and health care.^15,28^ Spouses often help remind patients of care times, encourage them to visit health centers, adherence to hygiene principles, and follow-up of treatment.^28^ In addition, married life can enhance motivation for preventive behaviors by increasing emotional support and creation of a sense of responsibility toward family health.^28^ Previous research in the field of infectious diseases, such as tuberculosis, has shown that married people tend to have more health-promoting behaviors and are more likely to participate in preventive measures compared to those who are single or lack family support.^15^ Therefore, it seems that social support resulting from marriage and increased motivation to protect one’s own health and that of family members can explain the positive relationship between marital status and tuberculosis preventive behaviors.

 The relationship between income level and TB preventive behaviors was not statistically significant in the multivariable regression model. However, a descriptive trend was observed suggesting that individuals with higher income may have slightly better preventive behaviors. Although this association did not remain significant after adjustment for other variables, socioeconomic status may still play an indirect role in shaping TB preventive behaviors through improved access to healthcare services, better living conditions, and greater availability of health-related information.^24^ These contextual factors may be particularly relevant in populations living in resource-limited settings.^24^

 The findings of our study showed a significant relationship between attitude and TB preventive behaviors. Attitude refers to an individual’s set of beliefs and evaluations regarding the outcomes of a behavior and can play an important role in the formation and maintenance of health-oriented behaviors.^16^ Individuals with a more positive attitude toward TB prevention usually perceive the risk of the disease more seriously and are more likely to accept the benefits of preventive actions; consequently, they show a greater tendency to engage in behaviors such as maintaining personal hygiene, timely visits to health centers, and adherence to treatment recommendations.^15,27^ Therefore, it appears that attitude, through its influence on an individual’s motivation and willingness to engage in protective actions, plays an important role in strengthening TB-related preventive behaviors.

 An additional finding of the SEM analysis was that tuberculosis-related knowledge was positively associated with both attitude and self-efficacy, suggesting that knowledge may be related to preventive behaviors through its associations with attitude, self-efficacy, and health literacy; however, this interpretation should be considered exploratory because formal mediation analysis was not conducted. This finding may explain why knowledge was significantly correlated with preventive behaviors in bivariate analyses but did not emerge as an independent predictor in the final regression model. Individuals who possess greater knowledge about TB transmission, symptoms, and prevention may develop more favorable attitudes toward preventive actions and greater confidence in their ability to engage in such behaviors, which subsequently facilitates behavioral adoption.

 In this study, health literacy was the strongest predictor of TB preventive behaviors. This finding is consistent with previous studies that have confirmed the key role of health literacy in promoting health-related behaviors.^18,23^ Individuals with higher levels of health literacy are better able to understand information related to the symptoms, modes of transmission, and methods of TB prevention and to apply health recommendations in their daily lives.^18^ In addition, higher health literacy enhances individuals’ ability to communicate effectively with healthcare providers, appropriately utilize health services, and follow preventive measures.^23^ Previous research has also shown that low health literacy is associated with less awareness of infectious diseases, delays in diagnosis, and lower adherence to health recommendations, whereas higher health literacy is associated with increased preventive behaviors and greater participation in disease control programs.^18,29^ Therefore, in line with the present study, individuals’ ability to understand and use health information can play an important role in the formation and strengthening of TB-related preventive behaviors.

 Contrary to expectations, self-efficacy did not demonstrate a significant direct association with TB preventive behaviors in the final structural model. One possible explanation is that the influence of self-efficacy may be associated with preventive behaviors in combination with other cognitive constructs, particularly health literacy and attitude; however, this possible pathway requires confirmation in future mediation studies. When these factors were simultaneously considered, the unique contribution of self-efficacy to preventive behaviors diminished. This finding highlights the dominant role of health literacy and attitude in shaping TB preventive behaviors and suggests that confidence alone may be insufficient to promote preventive actions unless accompanied by adequate understanding of health information and positive evaluations of preventive practices.

## Implications for Practice

 The findings of this study have important implications for tuberculosis control programs. First, interventions should prioritize improving health literacy as the most influential determinant of TB preventive behaviors. Educational programs should focus on enhancing individuals’ ability to understand and apply health information rather than solely increasing disease-related knowledge. Second, interventions should target high-risk populations, including individuals with chronic diseases, smokers, and immunocompromised patients, as these groups are more vulnerable to tuberculosis. Third, strengthening positive attitudes toward TB prevention through community-based education and healthcare provider engagement may further enhance preventive behaviors. Finally, integrating cognitive-based interventions into primary healthcare services may improve early detection and adherence to preventive practices.

## Strengths and Limitations

 Strengths of this study include the use of a relatively large sample size, application of multistage cluster sampling, and integration of both hierarchical regression and structural equation modeling, which allowed for a comprehensive assessment of predictors and hypothesized pathways among study variables.

 This study has several limitations that should be considered when interpreting the findings. First, the cross-sectional design limits the ability to establish causal relationships between cognitive factors and TB preventive behaviors. Second, the use of self-reported measures may introduce recall bias and social desirability bias, potentially leading to overestimation of preventive behaviors. Third, the study sample was limited to a specific population in Iran, which may restrict the generalizability of the findings to other cultural or geographical contexts. Fourth, test–retest reliability was not assessed for the measurement instruments; therefore, the temporal stability of the questionnaires could not be confirmed. Finally, despite controlling for demographic variables, the model explained a moderate proportion of variance in preventive behaviors, indicating that other unmeasured factors may also contribute to these behaviors.

 It should also be acknowledged that the study was based on a Knowledge–Attitude–Practice (KAP) framework, which is not a formal behavioral theory. Therefore, the integration of KAP with constructs such as self-efficacy and health literacy may not represent a fully established theoretical model. Future research is recommended to employ more comprehensive behavioral theories, such as the Multi-Theory Model (MTM) of health behavior change, to provide a stronger theoretical foundation.

## Conclusion

 This study identified key cognitive determinants of TB preventive behaviors while controlling for demographic characteristics. The findings demonstrated that health literacy was the strongest predictor of TB preventive behaviors, followed by attitude, highlighting the central role of individuals’ ability to understand, interpret, and apply health information in shaping preventive actions. Although demographic variables such as education level, marital status, and income showed associations with preventive behaviors in unadjusted analyses, only some of these relationships remained significant in the final regression model. Specifically, income level was not statistically significant after adjustment for cognitive factors.

 These findings suggest that cognitive factors may play a potential role in explaining the relationship between sociodemographic characteristics and TB preventive behaviors; however, this pathway was not formally tested using mediation analysis. Therefore, this interpretation should be considered exploratory and hypothesis-generating, requiring further investigation using appropriate mediation models in future research. These findings emphasize the importance of moving beyond purely biomedical approaches and focusing on cognitive and behavioral dimensions in TB control programs.

## Competing Interests

 Authors have no conflicts of interest to declare.

## Data Availability Statement

 The data that support the findings of this study are available from the corresponding author upon reasonable request.

## Ethical Approval

 Ethical approval for this study was obtained by the Ethics Committee of the Sarab Faculty of Medical Sciences (Ethics Code: IR.SARAB.REC.1402.011). Written informed consent was obtained from all participants prior to their inclusion in the study.
